# Targeted therapy in renal cell carcinoma: moving from molecular agents to specific immunotherapy

**DOI:** 10.1007/s00345-013-1033-3

**Published:** 2013-02-12

**Authors:** Jens Bedke, Cécile Gouttefangeas, Harpreet Singh-Jasuja, Stefan Stevanović, Carl-Ludwig Behnes, Arnulf Stenzl

**Affiliations:** 1 Department of Urology, Eberhard Karls University Tübingen, Hoppe-Seyler-Str. 3, 72076 Tübingen, Germany; 2 Department of Immunology, University of Tübingen, Tübingen, Germany; 3 Immatics Biotechnologies GmbH, Tübingen, Germany; 4 Department of Pathology, University of Göttingen, Göttingen, Germany

**Keywords:** Renal cell carcinoma, Tyrosine kinase inhibitor, Immune therapy, Vaccination, IMA901

## Abstract

Non-specific immunotherapy has been for a long time a standard treatment option for patients with metastatic renal cell carcinoma but was redeemed by specific targeted molecular therapies, namely the VEGF and mTOR inhibitors. After moving treatment for mRCC to specific molecular agents with a well-defined mode of action, immunotherapy still needs this further development to increase its accuracy. Nowadays, an evolution from a rather non-specific cytokine treatment to sophisticated targeted approaches in specific immunotherapy led to a re-launch of immunotherapy in clinical studies. Recent steps in the development of immunotherapy strategies are discussed in this review with a special focus on peptide vaccination which aims at a tumor targeting by specific T lymphocytes. In addition, different combinatory strategies with immunomodulating agents like cyclophosphamide or sunitinib are outlined, and the effects of immune checkpoint modulators as anti-CTLA-4 or PD-1 antibodies are discussed.

## Introduction

The observation of rare spontaneous tumor regressions in RCC has led to the early assumption that RCC is an immunogenic tumor [1]. Additionally, RCC tumors express higher levels of HLA class I and class II molecules compared to non-tumoral tissue [2, 3]. RCC tissue is frequently infiltrated by immune cells especially functional T lymphocytes [4, 5]. Therefore, strategies which harness the adaptive immune system were early considered as promising therapeutic options. Non-specific immunotherapy with the cytokines interleukin-2 (IL-2) and/or interferon-alpha (IFN-α) has been largely used in the past 25 years with the result of a notable clinical benefit (disease stabilization or remission) reported in up to one-third of treated patients. Long-term complete responders (CRs) are rare, but regularly observed [8]. However, median survival is only marginally enhanced, so non-specific immunotherapy is rarely used nowadays [6, 7]. In high-dose IL-2-treated patients, retrospective analyses proposed both high carbonic anhydrase IX and a pathologic risk classification based on extent of the alveolar morphology to forecast CR [8, 9]. These features were prospectively evaluated in the SELECT trial, but the predictive value of these putative biomarkers was not confirmed. Additionally, increased frequencies of regulatory T cells (T_reg_) and decreased frequencies of circulating myeloid and plasmacytoid dendritic cells have been reported in cytokine-treated mRCC patients and may partly explain the limitations of such therapy [10, 11].

### Targeted therapy

While enthusiasm for non-specific immunotherapies dampened, the discovery of the Von-Hippel–Lindau (VHL) gene and of its related molecular pathways and mechanisms built the basis for the era of “targeted” therapy [12]. Since 2005, different tyrosine kinase (TK) inhibitors targeting the VEGF receptor and mammalian target of rapamycin (mTOR) inhibitors have been successively introduced in the clinical routine for the treatment of mRCC patients [13]. Both median progression-free (PFS) and overall survival (OS) are substantially prolonged with these new substances, exceeding significantly the results obtained during the cytokine era. However, a profound prolongation of survival leading to long-term survivors has not been described so far. In addition, the prolongation of OS is compromised by drug-induced side effects which lead to dose interruption in up to 38 % of the patients [12, 14]. Due to this limited improvement of TK or mTOR inhibitors in the long-term, new therapy options are required to further improve patients’ cancer-specific survival (CSS).

Interestingly, it was observed that targeted agents do not only inhibit angiogenesis and tumor cell proliferation, but also show immunomodulatory effects directing the immune system to a stronger anti-tumor response [15]. For instance, sunitinib-treated mRCC patients show decreased frequencies of T_regs_ and myeloid-derived suppressor cells (MDSCs) in the peripheral blood [16, 17]. At the same time, sunitinib may shift T-helper cells toward a Th1-type response [16]. In contrast, sorafenib has immunosuppressive effects with a reduced induction of antigen-specific T cells in vitro and in immunized mice [15, 18]. Additionally, mTOR antagonists inhibit the calcineurin-dependent activation of the IL-2 gene transcription in response to T-cell receptor activation [19]. Therefore, combining the compatible targeted agents with immune therapy appears like a promising therapeutic option, especially if the non-specific immune stimulation can be redirected toward a more specific, efficient and durable adaptive immunity against tumor cells.

## Specific immunotherapy

Cytokine therapy with IL-2 and IFN-α non-specifically activates the immune system. This immune therapy does not present a very well-defined mode of action and does not induce a specific T-cell response directed toward known tumor-associated antigens (TAAs). Because of that, specific biomarkers or assays for immune monitoring of tumor-directed T cells cannot be available to monitor response to therapy. More importantly, due to its non-specific nature, the efficacy of such immunotherapy is limited, while the adverse events are substantial. It would be therefore highly desirable to activate effector T lymphocytes, especially cytotoxic CD8+ T cells, against tumoral, but not healthy tissues while inducing a long-lasting memory response against cancer cells. This can only be efficiently achieved by directing these T cells toward target structures specifically expressed or overexpressed in tumor cells.

### Tumor-associated antigens

**Fig. 1:**
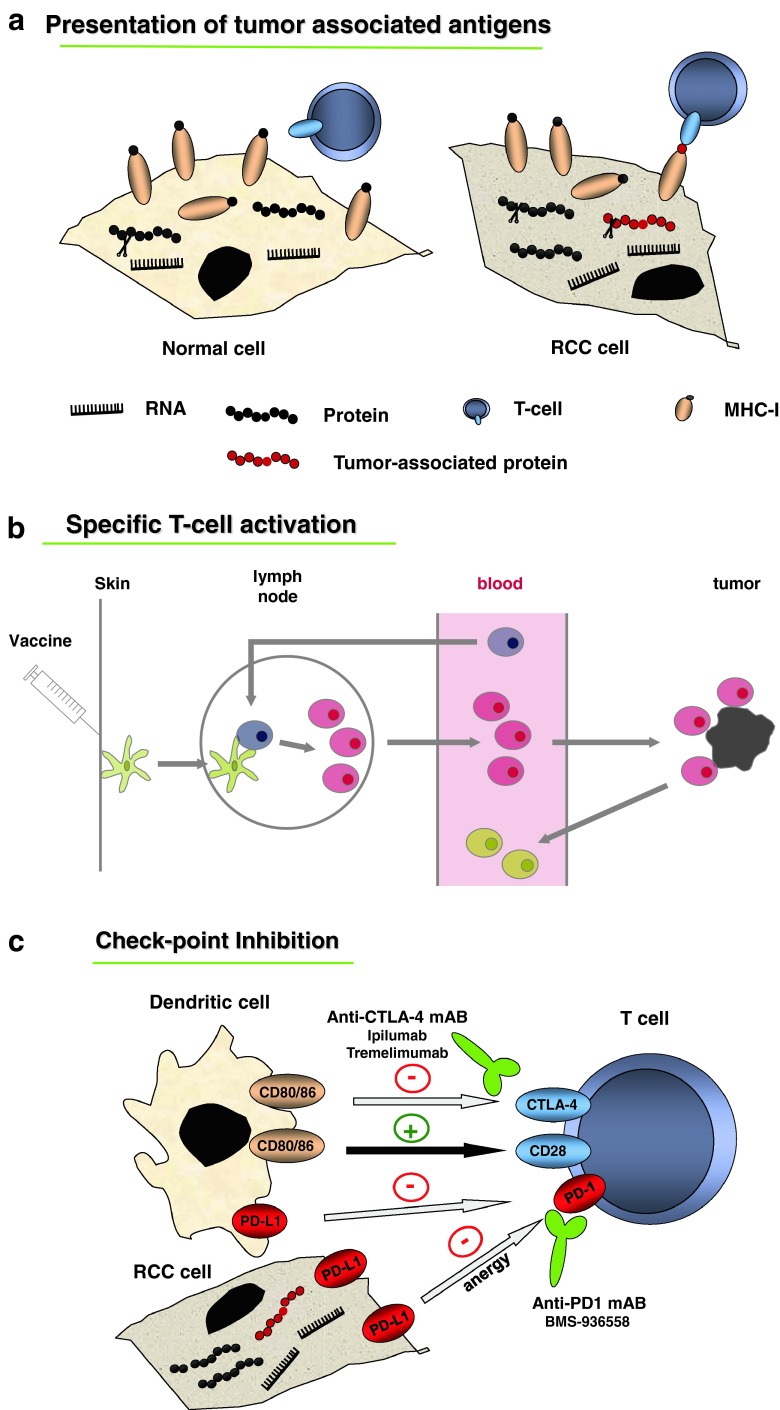
**a** Presentation of tumor-associated antigens. **b** Specific T-cell activation. **c** Immune checkpoints in RCC

It is well known that TAAs expressed by tumor cells can be very specifically recognized by the T-cell receptor (TCR) of cytotoxic CD8+ T lymphocytes. TCRs can bind specifically to short peptides of typically 8–10 amino acids in length derived from intracellular proteins and presented by human leukocyte antigen (HLA) molecules on the cell surface. Cell antigen processing leads to the display of such HLA-restricted peptides derived from TAAs, also known as tumor-associated peptides (TUMAPs). For generating TUMAPs, two main steps are necessary: First, the cleavage of the protein within the tumor cell by specific proteases must generate the peptide itself or a slightly longer precursor, and second, this peptide must contain a so-called HLA peptide motif for loading into the groove of the relevant HLA class I allele (Fig. 1a) [20]. Therefore, such HLA–peptide complexes represent suitable targets against which the host’s immune system can be activated in order to eliminate tumor cells.

In cancer vaccination, the choice of targeted TAAs is therefore crucial. The tumor specificity of cancer germ-line antigens (CTAs) is due to the fact that they are expressed in male germ cells and trophoblastic tissues, but not in other normal adult tissues except some tumor types. The cancer germ-line antigen MAGE-1 was the first TAA identified as a target of human CD8+ T cells and proved the concept of specific tumor recognition in patients [21]. Overexpressed antigens are those present in benign tissue, but at a significantly higher level on tumor cells. In RCC, carbonic anhydrase IX (CA IX) and Apolipoprotein L1 are such TAAs and appear particularly interesting since the presence of the T-cell epitopes derived from such TAAs has been confirmed in tumor tissue by analytical methods [22]. Another class of antigens results from mutated proteins. These are especially interesting for specific immunotherapy, since they are unique and solely expressed by the tumor cells. Mutation in the tumor protein sequence enables either a peptide to bind to HLA while the “wild-type” sequence does not or the new amino acid sequence induces a highly “mutation-specific” T-cell response. The great advantage of such mutated antigens lies in their true tumor specificity, potentially no triggering of peripheral tolerance and their frequent occurrence in driver genes decreasing the risk of immune escape due to the loss of expression [23]. Moreover, it can be assumed that specific T cells have not been subjected to T-cell deletion during thymus development and do not have been subjected to central tolerance. However, the uniqueness of TAA mutations may also be seen as a disadvantage, since they must be identified in each individual mRCC patient. However, recent technological progresses are paving the way to a fully individualized immunotherapy approach in the foreseeable future.

### Autologous tumor immunotherapy

Based on the knowledge that the immune system, for example, the cytotoxic CD8+ T cells, can be specifically directed against TAAs, different vaccines consisting of professional antigen-presenting cells [i.e., dendritic cells (DCs)] loaded with either allogeneic or autologous tumor-derived lysates or RNA with or without alongside administration of IL-2 were tested [24, 25].

The autologous tumor cell lysate vaccine Reniale^®^ (Liponova, Hanover, Germany) was evaluated in the adjuvant setting in high-risk RCC after nephrectomy. Five-year PFS was significantly improved as compared to observation (77.4 vs. 67.8 %), but the vaccine was not approved by the EMA for various reasons, including issues in trial methodology [26, 27]. Another adjuvant phase III trial assessed the efficacy of Vitespen, an autologous tumor-derived heat shock protein Gp96 preparation (Oncophage^®^. Antigenics Inc., Lexington, MA), and failed to reach the primary endpoint of PFS prolongation, despite some indication of activity in retrospectively defined subgroups [28].

Adoptive T-cell transfer represents another, considerably successful strategy in tumor immunotherapy. It is known that often tumor-infiltrating lymphocytes (TIL) are present within the RCC tumor tissue [4]. The presence of TILs suggests that the patient’s immune system has been activated to fight the tumor. Adoptive T-cell transfer of in vitro selected and/or expanded anti-tumor T cells is an approach to provoke a graft versus host reaction. The transplanted T cells attack the tumor which will be recognized, for example, as foreign tissue. Results of this approach are limited in RCC with no improvement of OS in phase I/II studies [29]. The genetic reprogramming of T cells has demonstrated promising preclinical data [30].

### Immune biomarkers

Tumor-based approaches such as those mentioned above are characterized by the use of a complex mixture of undefined proteins, potentially including many undefined TAAs presented by several HLA alleles. Such vaccines have the intrinsic limitation that the TAAs that are processed and then displayed on the surface of the APCs are different in the individual patients and therefore it is unknown against which TAA-specific T cells might be activated. Consequently, due to the unknown TAAs, monitoring of vaccine immunogenicity is inherently difficult and mostly incomplete. This is disappointing, since measurement of T cells or even antibodies is of critical importance to assess the effects of the vaccine on the immune system, to compare the efficacy of various clinical approaches and, hopefully in the near future, to predict therapy efficacy. To achieve these ambitious aims, in vitro immunomonitoring should be robust, reproducible and sensitive; however, up to now, no particular assay has been designed as being the gold standard to be applied. The Immunoguiding Program of the Association for Immunotherapy of Cancer (CIP/CIMT) is an international network working on the harmonization of the methods applied for in vitro monitoring of T cells among the community [31]. This and further initiatives should help to compare vaccine strategies and to accelerate progresses in the field [32, 33]. Meanwhile, several studies indeed demonstrate a correlation between vaccine immunogenicity measured in the blood and clinical benefit [34, 35].

### Single antigen-based tumor vaccines

Some more recent approaches have used defined single TAA. In the TROVAX renal immunotherapy survival trial (TRIST), a modified vaccinia Ankara vector engineered to deliver the tumor antigen 5T4 (MVA-5T4; TroVax™, Oxford BioMedica, Oxford, UK) was evaluated in a phase III study in combination with sunitinib, plus interleukin-2 or interferon-α. However, the primary endpoint of OS was not reached (median 20.1 vs. 19.2 months; TROVAX vs. placebo) [36]. One potential reason maybe that immune evasion by target down modulation which is more likely with a single antigen is being targeted [37].

### Multi-antigen peptide-based vaccination

Vaccination of RCC patients with synthetic peptides representing TAA-derived T-cell epitopes, that is, TUMAPs, presents several advantages: First, the manufacturing of synthetic peptides is relatively easy and cost-effective and they are very stable allowing long-term storage. Second, as shown by multiple vaccination trials in various cancer types so far, they are safe [34, 38]. Moreover, in vitro immunomonitoring of TUMAP-specific T cells is possible since the target structures recognized by the T cells are well defined. And fourth, the mixture of several peptides of different TAAs virtually allows covering a broad range of antigens with the result of a decreased risk of an immunological tumor escape.

Peptides can be either loaded ex vivo onto patient’s autologous DCs which will then be given back to the patient, or injected directly by intradermic or subcutaneous administration, where the peptides will be taken up by skin-resident DCs [25]. In theses approaches, optimal DCs activation and migration to the regional lymph node is crucial and can be supported by the administration of immunological adjuvants and/or immunomodulators (Fig. 1b).

A recent vaccine development following this principle for mRCC is IMA901, which consists of nine TUMAPs restricted by HLA-A*02 and one pan-HLA-DR-binding class II peptide. In two multicenter phase I and II trials, IMA901 was tested for safety, immunogenicity and T-cell response in association with a clinical benefit [34]. In the first phase I trial, 16 patients progressed, 11 had stable disease and 1 showed a partial response out of 28 patients enrolled after 3-month follow-up. None of the patients evaluable for the safety analysis experienced a drug-related severe adverse event. Among the 27 immune-evaluable patients those who responded to several TAAs, at least two were more likely to experience a clinical benefit. Additionally, low blood levels of regulatory T cells (CD4+ Foxp3+ T_regs_) before the start of therapy were correlated to a multiple T-cell response after vaccination [34].

A total of 68 patients of the phase II study had progressive disease after at least one previous cytokine and/or TKI pretreatment. Patients were randomized 1:1 to receive IMA901± a low-dose cyclophosphamide (Cy) pretreatment with the aim to reduce levels of T_regs_ and boost the immunological benefit of the vaccine. The rate of immune responders (64 %) was similar to that of the first trial, with 26 % of patients responding to more than one TAA. Although Cy did not improve the rate of immune responders, Cy-pretreated patients showed a prolonged median OS of 23.5 months (vs. 14.8 months without Cy). In a subgroup analysis of immune- and non-responders with or without the addition of Cy, only patients who exhibited an immune response to the vaccine showed a benefit from Cy pretreatment. This indicates that Cy has no single-agent activity but rather acts as an immunomodulator of the vaccine [34]. In this study, OS positively correlated with the number of induced T-cell responses. Nevertheless, the mechanisms to induce a multi-T-cell response could not be fully determined as the Cy approach did not increase peripheral immune responses.

This work is the first to demonstrate a clear association between an induced anti-TUMAP T-cell response and a clinical benefit measured as prolonged OS in mRCC. Moreover, it illustrates that a careful immunomonitoring can constitute a rational basis to modify therapies for improving patient’s benefit.

## Hurdles in peptide vaccination

Currently, there are several challenges in peptide vaccination: Which TAA should be targeted and in which format (long vs. short peptides, induction of CD8+ or CD4+ T-cell epitopes)? How can an efficient T-cell response in terms of dosage, route of administration and choice of adjuvants/immunomodulators be induced? And how can this response be sustained over time, for example, by the vaccination schedule?

### HLA restriction

A possible disadvantage of peptides is their HLA restriction limiting the eligibility of patients in clinical trials. However, with the advancement of formulation technologies, the next generation of peptide vaccines will be composed of peptides restricted to a number of the most common HLA alleles allowing coverage of greater than 90 % of the patient population. Currently, more and more non-HLA-A*02 TUMAPs are identified and tested in RCC for their immunologic response [39, 40]. Moreover, new technologies of next-generation sequencing coupled with improvements in mass spectrometry will allow the identification of the entire HLA ligandome, including mutation-derived sequences in individual patients in the near future. This will allow the use of TUMAPs derived from mutated tumor proteins which seems to be very robust in their presentation on the tumor cell surface [23].

### Choice of the immunomodulators/adjuvants

The adjuvant’s role is to enhance the immunogenicity of the administered vaccine and can be differentiated from systemic immunomodulators as the adjuvants are locally and temporally restricted co-administered with the TUMAPs [23]. For instance, skin-resident DCs shall be boosted in their antigen loading and presentation, activation, and migration to the draining lymph node. Locally co-administered Montanide and GM-CSF are commonly used to trigger these processes, but only few studies have directly compared these substances. In addition, the reported effects of the widely used GM-CSF are contradictory as it might enhance T-cell response, but was also shown to lower it if used at high systemic doses [23, 41, 42]. Currently, triggering through Toll-like receptors (TLRs) is a favored option, and we also observed that TLR7 stimulation by imiquimod seems to increase clinical response rate in prostate carcinoma peptide vaccination [38].

## Immunological checkpoints and combination strategies with immunotherapy

The tumor microenvironment has developed a plethora of strategies to impair T-cell activation and silence activated T cells, which also prevent an effective immune response after vaccination [43, 44]. Tumor-driven immune suppression includes the downregulation of HLA molecules and/or TAA, which leads to a decreased immunogenicity or the induction of suppressive cytokines like IL-10 or TGF-β [43]. In addition, these factors can recruit T_regs_, MDSCs or tumor-associated M2 macrophages, which in turn again act in an immunosuppressive manner [45, 46]. Therefore, agents called immunomodulators or checkpoint inhibitors, which counteract tumor-induced immunosuppression, may raise the efficacy of immunotherapy. Several checkpoints controlling T-cell activation are well known, and clinical application of inhibitors has already shown remarkable success in cancer treatment (Fig. 1c).

One costimulatory cascade essential for efficient T-cell activation is elicited by the binding of CD80 and CD86 on the DC to CD28 on the T cell which is inhibited if CD80/86 binds to the inhibitory molecule cytotoxic T-lymphocyte antigen 4 (CTLA-4) expressed on activated T cells (Fig. 1c). The antibodies tremelimumab and ipilimumab are directed against CTLA-4 and thereby re-activate effector T cells. Currently, ipilimumab is only approved as monotherapy for the treatment for metastatic melanoma [47]. In a phase II trial of mRCC patients, it was shown to induce a partial response in 5 of 40 patients [48].

Another immune escape mechanism uses the PD-1 receptor. Tumor cells can express PD ligand-1 to silence T-cell activation. A humanized anti-PD-1 antibody has already shown promising effects in a phase I study including mRCC patients and is currently evaluated in further studies in mRCC [49, 50].

LAG-3 (CD223) is expressed on activated T cells and is involved in the downregulation of antigen-induced TCR activation, negatively regulating T-cell function and homeostasis. As a soluble recombinant humanized form (IMP321, sLAG-3-Ig), it activates APCs through MHC class II signaling [51]. In a phase I mRCC trial, IMP321 induced a sustained CD8+ T-cell activation and increased the percentage of long-lived effector memory CD8+ T cells at doses above 6 mg which translated to a stable disease in 7 out of 8 patients [52].

To achieve the maximal benefit for mRCC patients, it is likely that immunotherapy will need to be combined with targeted agents, currently approved for first-line therapy, or with checkpoint blockers or immunomodulators. Bevacizumab, an anti-VEGF antibody, in combination with Interferon-α, is currently the clinically most advanced and the only approved combination with (non-specific) immunotherapy. Two phase III studies demonstrated an increased OS of the combination as compared to IFN-α alone (18.3 and 23.3 months) [53, 54].

Using checkpoint inhibitors, the PD-1 targeting antibody BMS-936558 in combinations of sunitinib or pazopanib plus anti-PD-1 is evaluated in a phase I study still recruiting patients [55].

A rather unexpected property of TKIs is their immunomodulatory effect. As an example, sunitinib does not only inhibit angiogenesis and cell proliferation in mRCC, but also decreases the number of T_regs_ and MDSCs while maintaining DC function [15–17]. One of the advantages in specific vaccination or immunotherapy is that these treatments are characterized by low to minimal side effects which makes it easy to combine these together without an exponential increase in side effects.

Due to the immunomodulatory effect, the combination with sunitinib is a most challenging approach if compared to other TKI. AGS-003 is a RNA-loaded dendritic cell-based vaccine demonstrating a PFS of 11.9 months in a phase II if combined with sunitinib. Immune monitoring of AGS-003-treated patients showed an expansion of tumor antigen-reactive CD28^+^ cytotoxic T lymphocytes and a decrease in T_regs_ [56]. For the multipeptide vaccine IMA901, a large phase III trial has finished the recruitment of 340 patients who were randomized in a 3:2 fashion to IMA901 plus sunitinib versus sunitinib alone in first-line advanced RCC patients. Based on the previous phases I/II, Cy and GM-CSGF were additionally applied as immunomodulators with IMA901. First results are expected in 2014 [57].

## Summary and future perspective

The evolution of immunotherapy in RCC has followed a general refinement from rather non-specific approaches such as cytokine treatment or tumor cell lysates to the use of well-defined and selected T-cell targets. Although promising clinical results have been achieved with peptide-based vaccination, both clinical benefit and accessibility for all patients, irrespective of their HLA allele combination, need to be further improved. Furthermore, therapy is limited due to the lack of prospective phase III studies. Peptide vaccination induces a well-defined T-cell response which can be monitored precisely and was shown to correlate with clinical benefit. The identification of TAAs expressed with a high tumor tissue specificity, the knowledge of such TAAs suitable for each individual patient, the adequate in vivo activation of DCs and anti-tumor T cells are major tasks for the next years.

Currently, the combination of an established first-line therapy, which ideally not only targets angiogenesis and cell proliferation, but also presents immunomodulatory activities, together with a peptide vaccine cocktail appears as a highly promising strategy in mRCC treatment. In addition, specific checkpoint inhibitors like PD-1 antibody hold the promise to boost the specific TAA-directed T-cell response. Here, peptide vaccination is the new kid on the block in mRCC treatment whose complex anti-tumor power is currently only visible like the tip of an iceberg.
